# The Mystery of Michelangelo Buonarroti’s Goiter

**DOI:** 10.5041/RMMJ.10237

**Published:** 2016-01-28

**Authors:** Davide Lazzeri, Donatella Lippi, Manuel Francisco Castello, George M. Weisz

**Affiliations:** 1 Plastic Reconstructive and Aesthetic Surgery, Villa Salaria Clinic, Rome, Italy;; 2 Center for Medical Humanities, Department of Experimental and Clinical Medicine, University of Florence, Florence, Italy;; 3 School of Humanities, University of New England, Armidale, NSW, Australia; 4 School of Humanities, University of New South Wales, Sydney, Australia

**Keywords:** Art, goiter, Michelangelo, sketch

## Abstract

Whilst painting the vault of the Sistine Chapel, Michelangelo Buonarroti left an autographical sketch that revealed a prominence at the front of his hyper-extended neck. This image was recently diagnosed as goiter. The poet Michelangelo in a sonnet dated 1509 described himself as being afflicted by goiter similarly to the cats in the northern Italian Lombardy, a region with endemic goiter. Several narratives extended this sonnet into a pathological theory. The analyses of Michelangelo’s works, however, his portraits and self-portraits, of poems and major biographies, have not indicated the likelihood of goiter. This investigation makes an attempt to assess the diagnosis on clinical as well as iconographical grounds.

## INTRODUCTION

Between 1508 and 1512, under the patronage of Pope Julius II, Michelangelo Buonarroti (1475–1564) was entrusted to paint the vault of the Sistine Chapel.^1^,^2^ Once in charge, the Master promptly asked for a high scaffold to be built, reaching to the ceiling of the Chapel.^1^,^2^ Michelangelo chose to paint lying on his back, with extended arms, looking upwards,^3^ a position confirmed in 1509 by an autographical sketch.^4^

The drawing reveals a hyper-extended neck, an image also showing a prominent larynx. Since the late twentieth century this swelling was considered to be a goiter.^5^–^7^ The aim of the present work was to interpret the condition found at the Master’s neck, based on physio-pathological considerations.

## MATERIAL

**Figure 1.**
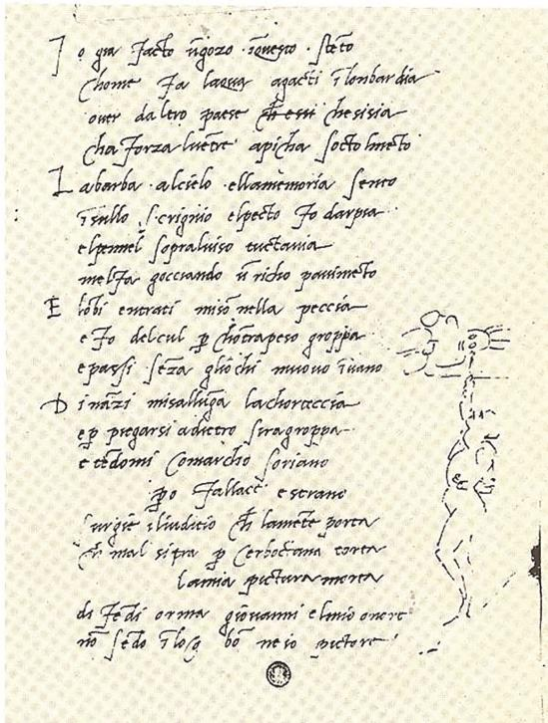
**Michelangelo Buonarroti.** Sonnet V (“Caudate sonnet”) of Michelangelo to his friend Giovanni da Pistoia and sketch by Michelangelo of himself painting the Sistine Chapel, 283 × 200 mm. (Fondazione Casa Buonarroti, Florence, Italy). Attribution: Michelangelo [Public domain], via Wikimedia Commons.

**Figure 2.**
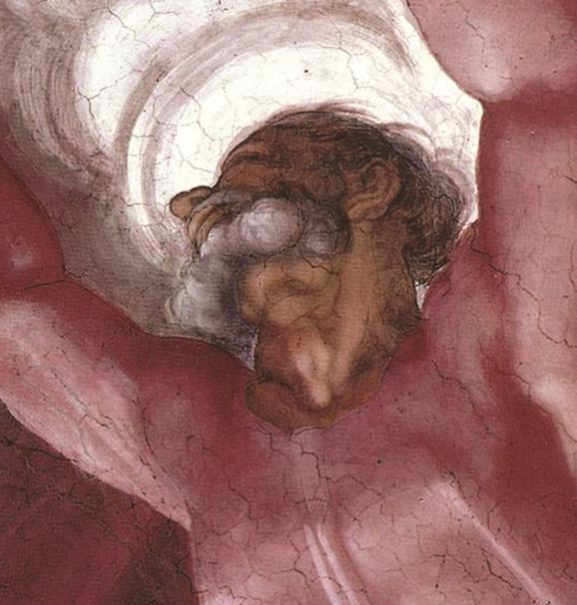
**Separation of Light from Darkness.** First of nine central panels frescoed along the center of the Sistine Chapel ceiling by Michelangelo in 1512. Close-up of the Creator. Sistine Chapel, Vatican, Vatican State. Attribution: Sistine Chapel, fresco Michelangelo [Public domain], via Wikimedia Commons.

Though Michelangelo wrote hundreds of poems, he did not entertain the thought of printing his poems in his lifetime; indeed, he distributed them freely amongst his friends. In the course of time some of his friends, partly by gifts and partly by obtaining copies, assembled a more or less complete collection.^1^–^3^ One sonnet, composed in 1509 (Box), was sent to his friend Giovanni da Pistoia and contained a drawing that constituted the subject of this investigation (Figure 1).^3^–^6^ The second portrait included in the present paper belongs to the first of nine central panels frescoed along the center of the Sistine Chapel ceiling by Michelangelo in 1512. The Master depicted a scene from the Book of Genesis in which God separated light from darkness (Figure 2).^5^

Box. **The “Sonnet V” (To Giovanni da Pistoia, *c.*1509), transl. S. Elizabeth Hall, *The Sonnets of Michelangelo Buonarroti*, p. 89 (1903).^3^**

**ON THE PAINTING OF THE SISTINE CHAPEL**

**In this hard toil I’ve such a goiter grown,**
**Like cats that water drink in Lombardy,**
**(Or wheresoever else the place may be)**
**That chin and belly meet perforce in one.**
**My beard doth point to heaven, my scalp its place**
**Upon my shoulder finds; my chest, you’ll say,**
**A harpy’s is, my paintbrush all the day**
**Doth drop a rich mosaic on my face.**
**My loins have entered my paunch within,**
**My nether end my balance doth supply,**
**My feet unseen move to and fro in vain.**
**In front to utmost length is stretched my skin**
**And wrinkled up in folds behind, while I**
**Am bent as bowmen bend a bow in Spain.**
**No longer true or sane,**
**The judgment now doth from the mind proceed,**
**For ’tis ill shooting through a twisted reed.**
**Then thou, my picture dead,**
**Defend it, Giovan, and my honour—why?**
**The place is wrong, and no painter I.**
**Michelangelo Buonarroti**

## METHODS

The first-mentioned sketch was found attached to the poem sent by Michelangelo to his friend Giovanni da Pistoia in 1509,^3^–^6^ a “caudate” sonnet (an expanded version of the standard sonnet form of 14 lines, followed by a *coda* or tail). In the first two stanzas of the poem, Michelangelo described himself as being afflicted by goiter, like cats in the northern Italian region of Lombardy, where goiter was endemic.^6^ Most likely, the feline reference may have been a metaphoric allusion to indicate north Italian peasants, who were nicknamed “cats” at the time of Michelangelo.^8^

A personality assessment with a retrospective diagnosis of a goiter ought to be based on parameters of today’s thyroid physiology and personality behavior. In thyrotoxicosis, the goiter in the front of the neck would be diffuse and smooth, slightly tender at times. This is accompanied by hyperactivity, restlessness, tachycardia, hypertension, sweating, heat intolerance, insomnia, loss of weight, hyper-metabolism, heart disease, and a shortened lifespan. Conversely, hypothyroidism would appear as a nodular, non-tender, prominent goiter, an over-weight body, intolerance to cold, apathy, slow reaction, somnolence, hypo-metabolism, with mental deficiency in adolescence. The personality differences are obvious.

## DISCUSSION

The words of the sonnet that initiated this line of diagnostic disputations indicate that Michelangelo was aware of the ancient Roman belief that some water (today’s iodine-deficient), was the cause of goiter.^7^,^9^ This was the case in the Alpine regions, in which the river water was addressed as “unhealthy for the throat,” or a possible cause for the “swollen throat.”^5^,^7^,^9^ This comment instigated a flow of hypothetical narratives that are interesting for interpreting the sketch and the fresco, although they are less scientific. Only a few are worthy of mentioning.

In their discussions, Martino and Mariotti argued that Michelangelo spent part of his youth in in a mountain valley of the Etruscan Apennines, a region known for endemic goiter. However, there is no evidence whatsoever that the Master, whilst living in his birthplace, exhibited any physical or mental retardation due to iodine deficiency. Indeed his physical prowess was eminent, and his intellectual development was appreciated at the Medici court when the Master was only in his teens. These two authors considered the Master’s goiter already present when he began painting the Sistine Chapel in Rome.^6^

This goiter hypothesis was further supported by Bondeson and Bondeson, interpreted based on the evidence that whilst working on the Sistine Chapel the artist made God in his own goitrous image (Figure 2).^5^ The authors of the present article found no evidence of such goiter in the fresco.

The imaginary theory was even further extended, connecting the external features of the neck with the internal neuro-anatomy of the cerebrum. Suk and Tamargo proposed that, in the *Separation of Light From Darkness*, Michelangelo drew in God’s neck a ventral view of the brainstem.^10^ The hypothesis that Michelangelo depicted himself as the goitrous Creator of the fresco is interesting but in our view not acceptable (Figure 2).

In 1509, at the time of the above-mentioned sonnet composition, Michelangelo was only 34 and was working long hours on a scaffold, with his neck hyper-extended. He was well and active. Assessed behaviorally, the Master was at times irritated, nervous, but there was no evidence of a hyperactive thyroid. It would be unrealistic to propose as a sign of thyrotoxicosis the single-minded work routine, unusual lifestyle, limited interests, poor social and communication skills, and issues of life control.^11^

Morphologically, in almost all portraits and self-portraits, Michelangelo is shown with a long beard. The Master painted three self-portraits as Jeremiah (on the ceiling of the Chapel), as the flayed skin figure displayed by Saint Bartholomew (in the Sistine Chapel’s “Last Judgment”), in the “Crucifixion of Saint Peter” (the Vatican’s Pauline Chapel), all with their neck covered, preventing any proper diagnosis of goiter.

**Figure 3.**
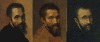
**Close-up of Michelangelo’s portraits.** **Left:** Portrait of Michelangelo Buonarroti (*c.*1535), by Jacopino del Conte, oil on panel (from the Casa Buonarroti Museum, Florence, Italy). **Center:** Portrait of Michelangelo Buonarroti (*c.*1544), by Daniele da Volterra, oil on wood (from the Metropolitan Museum, New York, US). **Right:** Portrait of Michelangelo Buonarroti (*c.*1595), by Pompeo di Giulio Caccini, oil on wood (from the Casa Buonarroti Museum, Florence, Italy). All panels ©Foto Scala Firenze, reprinted with permission of Scala Group S.p.a.

Michelangelo was also depicted by Raphael (as Heraclitus in *The School of Athens*, sitting on the front steps), and portraits were painted by Jacopino del Conte, Daniele da Volterra, and Pompeo di Giulio Caccini. They all portrayed the bearded Master in his later years of life, or copied after his death, similarly preventing any anatomical diagnosis. In our view, no goiter or neck swelling is visible in any of the aforementioned Michelangelo portraits (Figure 3).

Michelangelo wrote over three hundred sonnets and madrigals, and there is no mention of goiter other than in the above-mentioned “Sonnet V” sent to Giovanni da Pistoia. The Master lived for 89 years, and no sign of hyper- or hypothyroidism was described by contemporaries. His self-portraits, his activities, his long life—all would argue against any thyroid pathology.

Finally, our critical review of two major biographies (by Giorgio Vasari and Ascanio Condivi), as well as of his extensive correspondence, found no mention of goiter.^1^,^2^,^12^ It is also unlikely that the thermal water of Fiuggi, with which Michelangelo used to cure his nephrolithiasis, solved the goiter, as that water is not iodine-rich.^13^–^15^

## CONCLUSIONS

Initiated by a self-sketched image of Michelangelo and a description in his sonnet, a detailed hypothetical pathology of goiter emerged in the literature.

Analyzing the portraits and self-portraits as well as Michelangelo’s poems and two major biographies, reviewing his activities, and considering his longevity all suggest to us that active goiter was unlikely. Although the final diagnosis is speculative, the prominence at the neck, depicted by the Master himself, would be a prominent larynx, a positional condition, rather than a pathological one.
